# Competing endogenous RNA network analysis of Turner syndrome patient-specific iPSC-derived cardiomyocytes reveals dysregulation of autosomal heart development genes by altered dosages of X-inactivation escaping non-coding RNAs

**DOI:** 10.1186/s13287-023-03601-3

**Published:** 2023-12-20

**Authors:** Yumei Luo, Yapei Chen, Lingxia Ge, Guanqing Zhou, Yaoyong Chen, Detu Zhu

**Affiliations:** 1 Department of Obstetrics and Gynecology, Guangdong Provincial Key Laboratory of Major Obstetric Diseases, Guangdong Provincial Clinical Research Center for Obstetrics and Gynecology, Guangdong-Hong Kong-Macao Greater Bay Area Higher Education Joint Laboratory of Maternal-Fetal Medicine, The Third Affiliated Hospital of Guangzhou Medical University, Guangzhou, 510150 China; 2 Biologics Test and Evaluation Center, Guangzhou Laboratory, Guangzhou, 510005, China

**Keywords:** Turner syndrome, X chromosome, Induced pluripotent stem cell, Cardiomyocyte, Long non-coding RNA, Circular RNA

## Abstract

**Background:**

A 45,X monosomy (Turner syndrome, TS) is the only chromosome haploinsufficiency compatible with life. Nevertheless, the surviving TS patients still suffer from increased morbidity and mortality, with around one-third of them subjecting to heart abnormalities. How loss of one X chromosome drive these conditions remains largely unknown.

**Methods:**

Here, we have generated cardiomyocytes (CMs) from wild-type and TS patient-specific induced pluripotent stem cells and profiled the mRNA, lncRNA and circRNA expression in these cells.

**Results:**

We observed lower beating frequencies and higher mitochondrial DNA copies per nucleus in TS-CMs. Moreover, we have identified a global transcriptome dysregulation of both coding and non-coding RNAs in TS-CMs. The differentially expressed mRNAs were enriched of heart development genes. Further competing endogenous RNA network analysis revealed putative regulatory circuit of autosomal genes relevant with mitochondrial respiratory chain and heart development, such as COQ10A, RARB and WNT2, mediated by X-inactivation escaping lnc/circRNAs, such as lnc-KDM5C-4:1, hsa_circ_0090421 and hsa_circ_0090392. The aberrant expressions of these genes in TS-CMs were verified by qPCR. Further knockdown of lnc-KDM5C-4:1 in wild-type CMs exhibited significantly reduced beating frequencies.

**Conclusions:**

Our study has revealed a genomewide ripple effect of X chromosome halpoinsufficiency at post-transcriptional level and provided insights into the molecular mechanisms underlying heart abnormalities in TS patients.

**Supplementary Information:**

The online version contains supplementary material available at 10.1186/s13287-023-03601-3.

## Background

Adults with 45,X monosomy (Turner syndrome, TS) represent a minor population of survivors since more than 99% of these pregnancies terminate spontaneously during the first trimester [1]. The surviving individuals suffer from a wide spectrum of physical manifestations including short stature, gonadal dysgenesis, infertility, cardiovascular diseases, nervous system diseases and so on [2]. Among these symptoms, heart diseases are the leading reasons for the excessively early mortality of TS patients, likely shortening the life expectancy for more than 10 years [3]. Haploinsufficiency of genes escaping X-inactivation is a possible reason for the above symptoms, e.g., SHOX for short stature [4], PRKX for congenital urinary malformations, KDM6A for premature ovarian failure and TIMP1 for aortic aneurysm formation [5]. However, the genetic causes that drive most other symptoms, especially for the life-threatening heart diseases, remain largely unknown, thus requiring more systematic approaches to identify the hidden factors from other perspectives.

A recent study integrating transcriptome, DNA methylome and chromatin conformation analyses has demonstrated that haploinsufficiency of the transcriptional factor ZFX that escapes from X-inactivation is one of the key drivers for transcriptional dysregulation of autosomal genes in TS cells, which is termed “genomewide ripple effect” [6]. However, the investigation was based on peripheral blood mononuclear cells (PBMCs), and the analyses were focused on transcriptional factors. Hence, the global ripple effects due to the X haploinsufficiency at post-transcriptional level and during the developmental processes, especially for the heart development, are yet to be characterized systematically.

More and more evidences have supported that non-coding RNAs (ncRNAs) play important roles of post-transcriptional regulation in cardiomyocyte (CM) differentiation [7]. The long non-coding RNAs (lncRNAs) and circular RNAs (circRNAs) could serve as microRNA (miRNA) sponges and then indirectly regulate the expression levels of developmental genes via the “lnc/circRNA-miRNA-mRNA” regulatory circuits, which consist of the competing endogenous RNA (ceRNA) network [8]. Therefore, it is rational to hypothesize that haploinsufficiency of X-inactivation escaping lnc/circRNAs might drive a “genomewide ripple effect” via the ceRNA network during CM differentiation as well.

The emergence of induced pluripotent stem cell (iPSC) technology has provided an excellent tool for studying the molecular mechanisms underlying the human development. By using human iPSCs and their derived CMs (iPSC-CMs) as in vitro cellular models, the previous studies have identified a large number of lnc/circRNAs with regulatory functions during the CM differentiation [9–13]. Nevertheless, these CM-associated lnc/circRNAs are on autosomes and thus could not explain the cardiovascular abnormalities in TS patients. Therefore, whether haploinsufficiency of X-inactivation escaping lnc/circRNAs causes dysregulation of heart development genes via ceRNA network in TS-CMs still needs further investigation.

To circumvent these questions, we have established a panel of well-characterized TS patient-specific iPSC lines (TS-iPSCs) and developed a three-step protocol for generating beating CMs from iPSCs. Furthermore, we have profiled mRNA, lncRNA and circRNA expression in wild-type (WT)- and TS-iPSC lines and their derived CMs via high-density microarrays, and then performed ceRNA network analysis. As a result, we have identified a number of novel “lnc/circRNA-miRNA-mRNA” regulatory circuits for autosomal heart development genes mediated by X-inactivation escaping lnc/circRNAs.

## Methods

### Ethics

The study was conducted in accordance with the Declaration of Helsinki and approved by the Ethics Committee of The Third Affiliated Hospital of Guangzhou Medical University (Protocol no. 2017/058, 19 October 2017). Informed consents were obtained from the donors of PBMCs.

### PBMC isolation

Blood samples were collected from the donors into the BD Vacutainer Cell Preparation Tubes, and PBMCs were isolated by centrifugation at 1800×*g* for 30 min. The PBMCs were then transferred to a fresh tube, washed with PBS and centrifuged at 300×*g* for 15 min. The cell pellet was resuspended in 1-mL X-VIVO Hematopoietic Media (LONZA) and transferred to a 12-well plate.

### Generation of integration-free iPSC

The PBMCs were expanded and reprogrammed using the CytoTune-iPS 2.0 Reprogramming Kit (Invitrogen). Briefly, PBMCs were transduced with Sendai viruses expressing the reprogramming factors in X-VIVO media supplemented with EPO (2 U/mL), IGF-1 (40 ng/mL), SCF (50 ng/mL), IL-3 (10 ng/mL) and dexamethasone (1.5 μM). On day 3, the cells were trypsinized, and 5 × 10^5^ cells were seeded onto a Matrigel-coated 10-cm culture dish in mTeSR medium (STEMCELL Technologies). The medium was changed every day. In days 16–20, hESC-like colonies were picked mechanically and expanded in mTeSR medium on Matrigel (BD). Cells were routinely passaged every 4–5 days, and the medium was changed every day.

### Alkaline phosphatase (AP) staining and immunostaining

For AP staining, colonies were fixed with 90% alcohol for 2 min, washed three times with Tween-BST solution (PBS with 1% bovine serum albumin and 0.2% Tween-20) and then stained with BCIP/NBT (alkaline phosphatase substrate solution, Maxim Biotech) for 30 min in the dark. They were then washed with PBS twice and observed under the microscope.

For immunostaining, cells were fixed in 4.0% paraformaldehyde for 20 min, permeabilized with 0.5% Tween-20 in PBS, incubated with primary antibody overnight and then incubated with secondary antibodies (Invitrogen) for 1 h. Imaging was performed using an inverted confocal microscope. Primary antibodies used in this study were SSEA-4 (1:100, R&D), TRA-1-60 (1:200, R&D), OCT4 (1:500, R&D), SOX2 (1:500, R&D), AFP (1:500, R&D), nestin (1:100, R&D), SMA (1:500, R&D) and TNNT2 (1:500, R&D). 4, 6-Diamidino-2-phenylindole (DAPI) was used for nuclear staining.

### Karyotyping analysis

For cytogenetic analysis, cells were incubated in culture medium with 0.25-g/mL colcemid (Gibco) for 3 h, harvested, incubated in 0.4% sodium citrate, 0.4% chloratum:kaliumat (1:1, v/v) at 37 °C for 5 min and then fixed three times in methanol:acetic acid (3:1, v/v). After Giemsa staining, at least 100 splitting cells were examined for karyotyping.

### In vitro differentiation of iPSCs

The iPSC colonies were dissociated from Matrigel-coated dish with 1-mg/ml dispase (Gibco) treatment. The suspension was cultured on bacterial culture plates in embryonic body (EB) culture medium to allow aggregation and prevent adherence to the plate. The EB culture medium was changed every other day. After 5 days of suspension culture, formation of EB was examined. Then, the EBs were transferred to 0.1% gelatin-coated culture dishes for spontaneous differentiation. After 14 days of differentiation, immunostaining was performed using antibodies against α-fetoprotein (AFP, endoderm marker), smooth muscle actin (SMA, mesoderm marker) and nestin (ectoderm marker).

### Teratoma formation of iPSCs

The iPSC colonies at passage 10 were treated with dispase (1 mg/mL, Gibco) for 10 min at room temperature and then dispensed into 300–400 small colony suspensions. The colonies were collected and subcutaneously injected into the inguinal grooves of 6-week-old female SCID mice (two mice per cell line). Eight weeks later, the resultant tumors were removed, fixed in 4% paraformaldehyde for 4–8 h and embedded in paraffin. After staining with hematoxylin and eosin, the sections were examined for the presence of tissues derived from the three germ layers under a light microscope.

### Cardiomyocyte (CM) differentiation

TS-iPSCs were cultured on a Matrigel-coated 12-well plate in mTeSR medium. When the iPSCs reached 10–30% confluence, the culture media were changed to Essential 8 Medium (Gibco) and replaced with fresh media every day. On day 4, the culture media were changed to Cardiomyocyte Differentiation Medium A (Gibco). On day 6, the culture media were changed to Cardiomyocyte Differentiation Medium B (Gibco). On day 8, the culture media were changed to Cardiomyocyte Maintenance Medium (Gibco) and replaced with fresh medium every other day. On day 12, to enrich the CMs, the culture media were switched to Cardiomyocyte Enrichment Medium (RPMI 1640 Medium without glucose, with 7.5% Bovine Albumin Fraction V 3.3 mL, 60% Sodium Lactate Syrup 0.4 mL, 250 × Ascorbic Acid Solution 0.13 mL) and replaced with fresh medium every other day until day 16. Undifferentiated cells were lost substantially by metabolic selection during procedure. The efficacy of CM differentiation was evaluated by immunostaining for SMA and TNNT2. The CM beating frequencies of each cell line were calculated with at least four CM clusters. A total of three batches of CMs were generated from each group of iPSCs for the subsequent assays.

For gene knockdown treatment, the WT-iPSCs were transduced with lentiviral vectors (Cyagen Biotech) expressing shRNA-Nonsense-Control (sh-NC) and shRNA-lnc-KDM5C-4:1 (sh-Lnc), respectively. Then, the transduced iPSCs were differentiated into CMs as described above.

### Profiling mRNA, lncRNA and circRNA expression by microarray

The mRNA, lncRNA and circRNA expressions were profiled by Agilent ceRNA expression arrays. In brief, total RNA samples were collected from iPSCs and CMs at day 14 post-induction and checked for the RIN values to confirm RNA integrity by Agilent Bioanalyzer 2100. Then, the total RNA samples were amplified and labeled by the Low Input Quick Amp Labeling Kit, One-Color (Agilent Technologies). Labeled cRNAs were purified by the RNeasy Mini Kit (QIAGEN).

For microarray hybridization, each slide was hybridized with 1.65-μg Cy3-labeled cRNA using Gene Expression Hybridization Kit (Agilent) in Hybridization Oven (Agilent). After 17-h hybridization, slides were washed in staining dishes (Thermo Shandon) with the Gene Expression Wash Buffer Kit (Agilent).

Finally, the microarray slides were scanned by the Agilent Microarray Scanner with default settings: dye channel = green, scan resolution = 3 μm, PMT 100%, 20 bits. Data were extracted with the Feature Extraction software 10.7 (Agilent). Raw data were normalized by the Quantile algorithm of limma packages in the R software. The microarray data were deposited in the Gene Expression Omnibus (GEO) database under the accession no. GSE239758.

### Differentially expressed gene identification

After normalization by the limma packages, the differentially expressed (DE) mRNAs, lncRNAs and circRNAs were identified with criteria of |Fold Change (linear)|≤ 0.5 or |Fold Change (linear)|≥ 2 and *P* value < 0.05.

### Construct of co-expression network and ceRNA network

Firstly, the miRNAs targeting the DE mRNAs, lncRNAs and circRNAs was predicted by miRanda and TargetScan. Next, the co-expression network was built based on the Pearman’s correlation coefficient (PCC) analysis according to the expression levels of the genes. For each putative lnc/circRNA-miRNA-mRNA regulatory circuit, lnc/circRNA-mRNA pairs with PCCs greater than 0.2 and *P* < 0.05 were retained to construct the ceRNA network. Finally, the co-expression network and ceRNA network were visualized by the Cytoscape v3.6.0 software.

### Quantitative real-time PCR

For quantification of mRNA, lncRNA and circRNA expression levels, total RNA was extracted using TRIzol (Invitrogen) according to the manufacturer’s protocol. First-strand cDNA was synthesized using the TaKaRa PrimeScript II 1st Strand cDNA Synthesis Kit (TaKaRa). About 1 μL of cDNA reaction mix was subjected to PCR amplification using the SYBR Premix Ex Taq II Kit (TaKaRa) in the Bio-Rad CFX96 Real-Time PCR System (Bio-Rad). GAPDH was selected as the internal reference gene. For quantification of miRNA expression levels, small RNA was extracted using PureLink microRNA Isolation Kit (Invitrogen) and treated with TURBO DNA-free DNase (Ambion). Then, polyA tailing and cDNA synthesis were performed using the Ncode VILO microRNA cDNA Synthesis Kit (Invitrogen). U6 was selected as the internal reference gene. The primers used for qPCR analysis are listed in Additional file 8: Table S5.

### Mitochondrial DNA copy number assay

The DNA samples were purified from WT- and TS-CMs at day 14 post-induction. Mitochondrial DNA content was measured via qPCR targeting the mitochondrial gene 16S-RNA and normalized against the genomic gene GAPDH. A total of three batches of iPSC-CMs were evaluated. The primers used for this assay are listed in Additional file 8: Table S5.

### Statistical analysis

The results are represented as mean ± s.d. The statistical significance of differences was determined by paired, two-tailed Student’s *t*-test. A *P* value < 0.05 was considered to be statistically significant.

## Results

### Generation of integration-free iPSC lines from WT and TS female adults

**Fig. 1:**
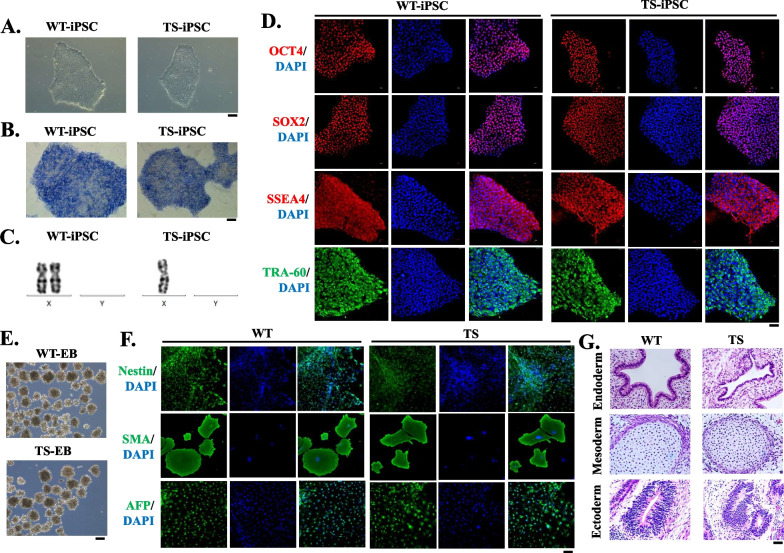
Characterization of wild-type (WT) and Turner syndrome (TS)-specific induced pluripotent stem cell (iPSC) lines. **A** Representative colony morphology of WT- and TS-iPSCs. **B** Both WT- and TS-iPSC colonies were positive for alkaline phosphatase staining. **C** Karyotype analysis showing X chromosome number of WT- and TS-iPSCs. **D** Immunofluorescence of the pluripotency markers OCT4, SOX2, SSEA4 and TRA-60 in WT- and TS-iPSCs. **E** Embryoid body (EB) differentiation of WT- and TS-iPSCs in vitro. **F** Immunofluorescence of germ layer markers nestin, SMA and AFP in WT- and TS-iPSCs. **G** Teratoma formation assay showing both WT- and TS-iPSCs differentiates into tissues of the three germ layers in vivo. Scale bar = 100 μm

Peripheral blood mononuclear cells (PBMC) were isolated from three healthy donors and three TS patients, respectively. Three WT-iPSC and three TS-iPSC lines were generated from the PBMCs by non-integrating Sendai viral vectors expressing Oct3/4, Sox2, c-Myc and Klf4. Two weeks after viral infection, a small number of colonies with typical morphology of human embryonic stem cell (hESC) emerged (Fig. 1A). We found that adding 4 μg/mL of Polybrene to the medium (suggested in CytoTune-iPS 2.0 Sendai Virus Kit manual) at the time of transduction did not increase transduction efficiencies in our hands, probably due to the intrinsic nature of the 45,X cells. All hESC-like colonies were picked manually and expanded clonally. All WT-iPSC lines showed 46,XX karyotype and TS-iPSC lines 45,X karyotype (Fig. 1C; Additional file 1: Fig. S1).

Both WT- and TS-iPSC lines exhibited strong alkaline phosphatase (AP) activity (Fig. 1B) and expressed human pluripotent stem cell markers SSEA-3, SSEA-4, TRA-1-60, TRA-1-81, OCT4, SOX2 and NANOG (Fig. 1D). All iPSC lines can form embryoid bodies (EBs) (Fig. 1E), and when plated in suspension, the EBs can spontaneously differentiate into cell types representative of the three embryonic germ layers, which were positive for AFP (endoderm marker), SMA (mesoderm marker) and nestin (ectoderm marker) immunostaining (Fig. 1F). Teratomas were observed 4–5 weeks after the iPSC lines were injected into SCID mice. Histological examination revealed that the teratomas contained the tissues derived from three embryonic germ layers, including glandular tissue (endoderm), cartilaginous tissue (mesoderm) and neural tube-like cells (ectoderm) (Fig. 1G).

### CMs derived from TS-iPSCs exhibit phenotypes of lower beating frequency and higher mitochondrial DNA copies

**Fig. 2:**
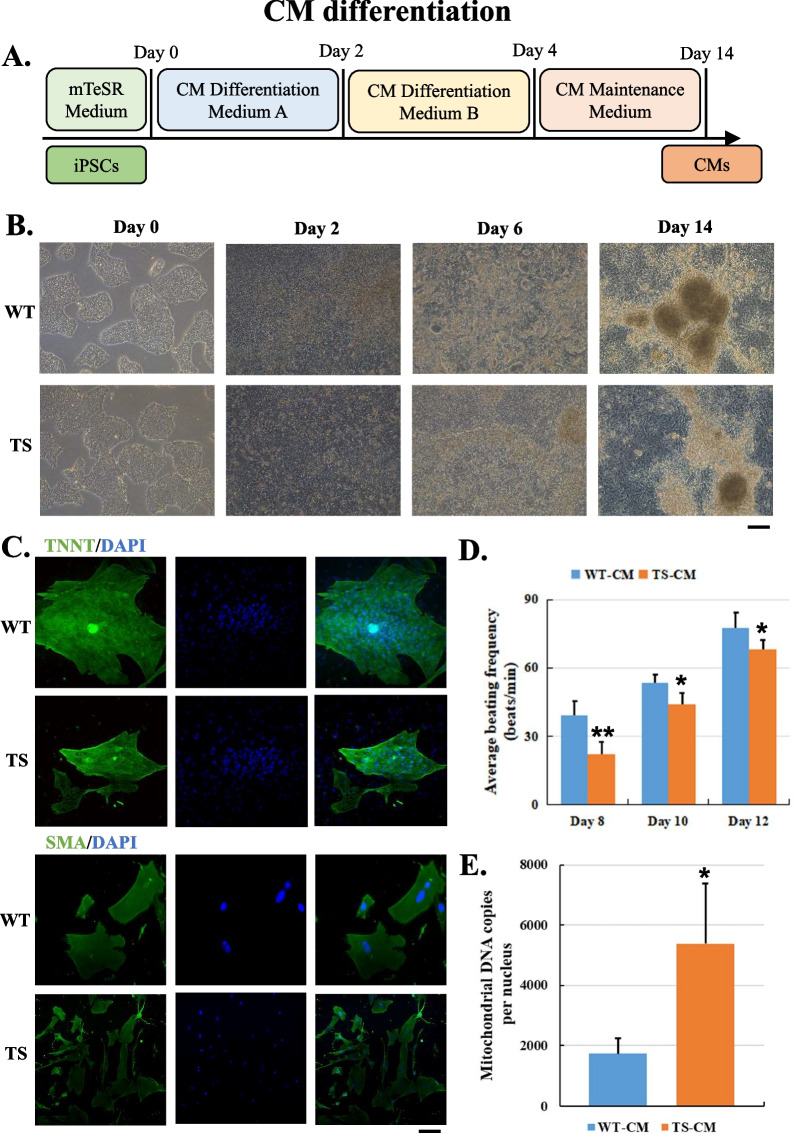
Differentiation of cardiomyocytes (CM) from WT- and TS-iPSC lines and characterization of iPSC-CMs. **A** A scheme of the protocol for iPSC differentiation into CM. **B** Cell morphology changes during iPSC-CM differentiation for 2, 6 and 14 days. Scale bar = 100 μm. **C** Immunofluorescence of CM-specific markers TNNT and SMA. Scale bar = 100 μm. **D** Average beating frequency of CMs at 8, 10 and 12 days. A total of three batches of CMs were generated from iPSCs, and four CM clusters of each batch were evaluated. **E** Average mitochondrial DNA copy number per nucleus of CM at 14 days. A total of three batches of iPSC-CMs were evaluated. Error bars indicate s.d.; *P < 0.05; **P < 0.01

The iPSC lines were expanded to 30–70% confluence before CM differentiation. We found that if the confluence of the cultures is outside this range, the CM differentiation will be less efficient. The iPSC lines underwent CM differentiation with a three-step protocol (Fig. 2A). As early as day 6, contracting cells could be observed; and on day 14, averagely 30% of the cells were spontaneously contracting (Fig. 2B). We compared the purification efficacy of several methods and found that adding growth factors greatly enhanced CM generation and enrichment with > 70% cardiac cells positive of TNNT and SMA staining (Fig. 2C). The beating frequencies of the TS-CMs were significantly lower than those of the WT-CMs (Fig. 2D; Additional file 2: Video S1 and Additional file 3: Video S2). In addition, the mitochondrial DNA copies per nucleus of the TS-CMs were almost onefold higher that those of the WT-CMs (Fig. 2E). It seemed that the mitochondrial bioenergetic function might be weaker in TS-CMs; thus, the beating frequency of TS-CMs was lower, and mitochondrial fission was induced in TS-CMs by a negative feedback loop.

### Comprehensive profiling of mRNA, lncRNA and circRNA expression in the WT- and TS-iPSCs and their derived CMs

**Fig. 3:**
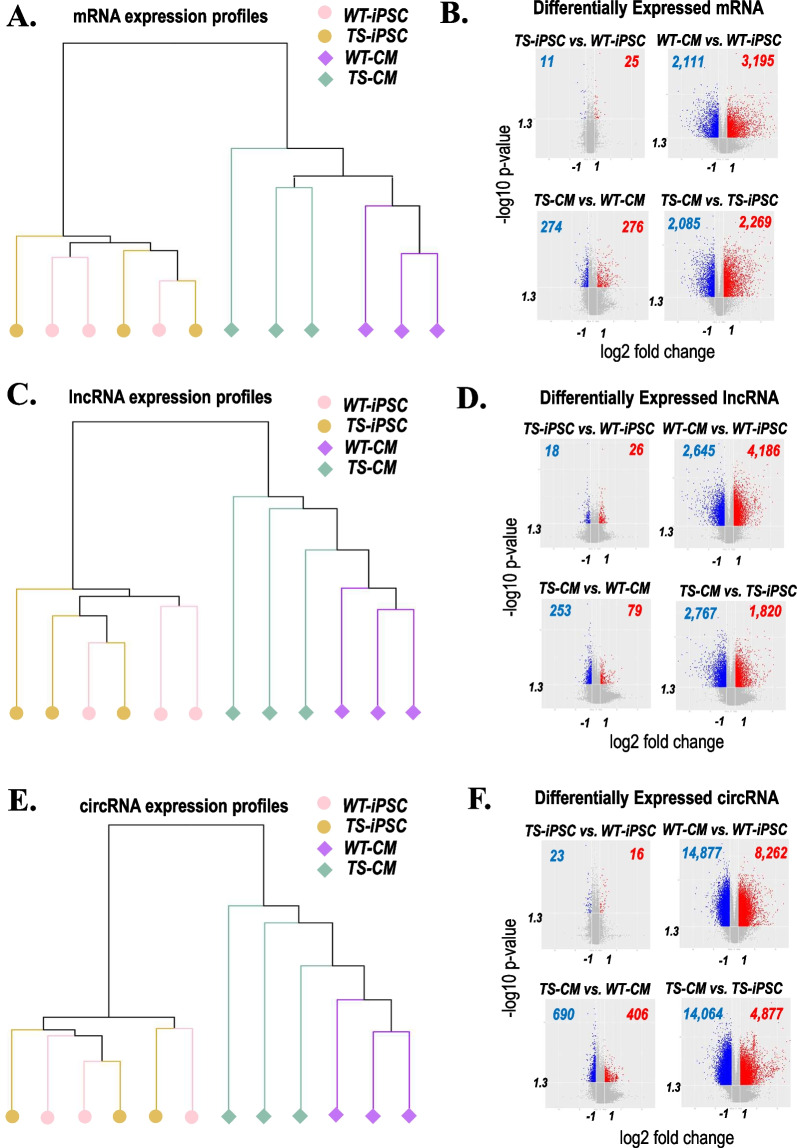
Comprehensive mRNA, lncRNA and circRNA expression profiling of WT- and TS-iPSCs and CMs. **A** Hierarchical clustering analysis of mRNA expression profiles among the samples of WT-iPSC, WT-CM, TS-iPSC and TS-CM groups. **B** Volcano plots showing differentially expressed (DE) mRNAs in group pairwise comparison. **C** Hierarchical clustering analysis of lncRNA expression profiles among the samples of all groups. **D** Volcano plots showing DE lncRNAs in group pairwise comparison. **E** Hierarchical clustering analysis of circRNA expression profiles among the samples of all groups. **F** Volcano plots showing DE circRNAs in group pairwise comparison

Total RNA samples were collected from all iPSC lines and their derived CMs on day 14 post-induction. The mRNA, lncRNA and circRNA expressions were profiled by microarray. Hierarchical clustering analyses showed similar clustering pattern for all the three classes of RNAs (Fig. 3A, C and E). Firstly, all the iPSC lines and all the CM lines were separated, which meant that the transcriptional alteration induced by cell differentiation was much larger than that induced by loss of a whole X chromosome (Fig. 3A, C and E; Additional file 1: Fig. S2). Secondly, the transcriptome differences between the iPSC lines were much less than those between the CM lines (Fig. 3A, C and E; Additional file 1: Fig. S2). Especially, all the WT-CM lines and all the TS-CM lines were clearly separated (Fig. 3A, C and E; Additional file 1: Fig. S2). It implicated that the global transcriptome alteration induced by loss of one X chromosome was more substantial in the differentiated CMs than in the undifferentiated iPSCs.

This observation is also supported by the number of differentially expressed (DE) genes identified by group pairwise comparison for all the three classes of RNAs (Fig. 3B, D and F; Additional file 4: Table S1, Additional file 5: Table S2 and Additional file 6: Table S3). There were largest sets of DE genes by WT-CM versus WT-iPSC and TS-CM versus TS-iPSC (Fig. 3B, D and F; Additional file 4: Table S1, Additional file 5: Table S2 and Additional file 6: Table S3). The DE genes by TS-CM versus WT-CM were lesser and those by TS-iPSC versus WT-iPSC the least (Fig. 3B, D and F; Additional file 4: Table S1, Additional file 5: Table S2 and Additional file 6: Table S3).

Taken together, the results implicated that: (1) The transcriptome alterations introduced by loss of a whole X chromosome are even less than those by cell differentiation program, which could partly explain why X monosomy is the only survivable monosomy. (2) The impacts of X monosomy on the global transcriptome were various in different cell types, from mild in iPSCs to strong in differentiated CMs.

### Differentially expressed mRNAs were enriched in heart development genes

**Fig. 4:**
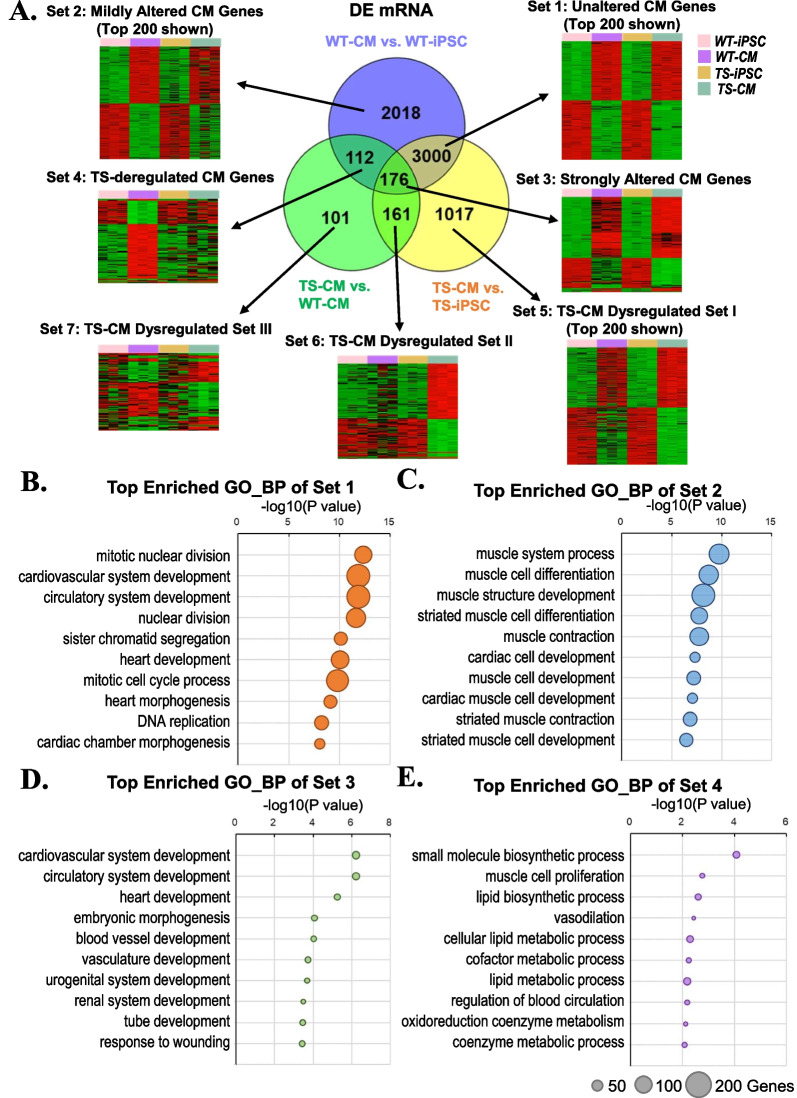
Identification of DE mRNAs and gene annotation enrichment analysis. **A** Venn diagram and heatmaps showing the numbers of DE mRNAs in different comparison groups. **B** Gene annotation enrichment analysis of DE mRNAs in Set 1. **C** Gene annotation enrichment analysis of DE mRNAs in Set 2. **D** Gene annotation enrichment analysis of DE mRNAs in Set 3. **E** Gene annotation enrichment analysis of DE mRNAs in Set 4

Next, we further looked into the DE mRNAs from group pairwise comparison. There were 3000 common DE mRNAs (Set 1) of both WT- and TS-CMs compared to their original iPSCs, which was the largest subset of DE mRNAs and represented for the CM-regulated genes unaltered by loss of one X chromosome (Fig. 4A). Gene ontology (GO) term enrichment analysis showed that these genes were enriched in mitotic nuclear division and cardiovascular system development (Fig. 4B; Additional file 7: Table S4.1). This is consistent with the observation that TS-iPSCs were capable of differentiating into CMs.

Regarding the altered CM-regulated genes, 2018 genes were mildly altered (Set 2), 176 strongly altered (Set 3) and 112 deregulated (Set 4). Overall, the majority of CM-regulated genes were unaltered or mildly altered, and only a small proportion were strongly altered or deregulated by loss of one X chromosome (Fig. 4A). GO analyses showed that the Set 2 mildly altered CM genes were enriched in contractile fiber of the muscle cells (Fig. 4C; Additional file 7: Table S4.2), and Set 3 strongly altered CM genes were enriched in vasculature (Fig. 4D; Additional file 7: Table S4.3). The Set 4 deregulated CM genes contained the fewest but some important CM genes, including COQ10A of the mitochondrial respiration chain, as well as WNT2 and FGF9 of the WNT signaling pathway (Fig. 4E; Additional file 7: Table S4.4). These altered and deregulated CM genes might partly explain the abnormal phenotypes of TS-CMs.

On the other hand, the X chromosome loss also induced dysregulation of around 1000 non-CM genes in TS-CMs (Fig. 4A, Sets 5–7). These genes were not significantly associated with important CM functions (Additional file 7: Tables S4.5–4.7).

### Construct of DE lncRNA-mRNA co-expression network

**Fig. 5:**
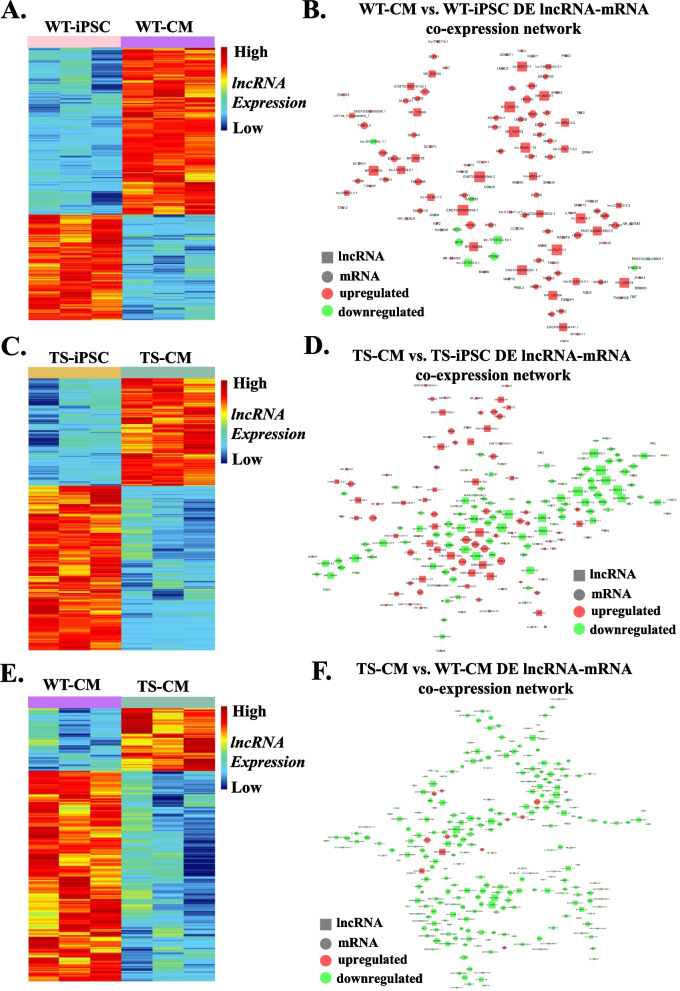
Identification of DE lncRNAs and co-expression network analysis. **A** Heatmap showing the DE lncRNAs in WT-CM versus WT-iPSC. **B** Co-expression network of DE lncRNA-mRNA in WT-CM versus WT-iPSC. **C** Heatmap showing the DE lncRNAs in TS-CM versus TS-iPSC. **D** Co-expression network of DE lncRNA-mRNA in TS-CM versus TS-iPSC. **E** Heatmap showing the DE lncRNAs in TS-CM versus WT-CM. **F** Co-expression network of DE lncRNA-mRNA in TS-CM versus WT-CM

Co-expression networks were constructed according to the correlation analysis of the DE lncRNAs and mRNAs in the group pairwise comparison (Fig. 5A, C and E). There were 288, 159 and 332 lncRNA-mRNA co-expression pairs identified for WT-CM versus WT-iPSC, TS-CM versus TS-iPSC and TS-CM versus WT-CM, respectively (Fig. 5B, D and F).

**Fig. 6:**
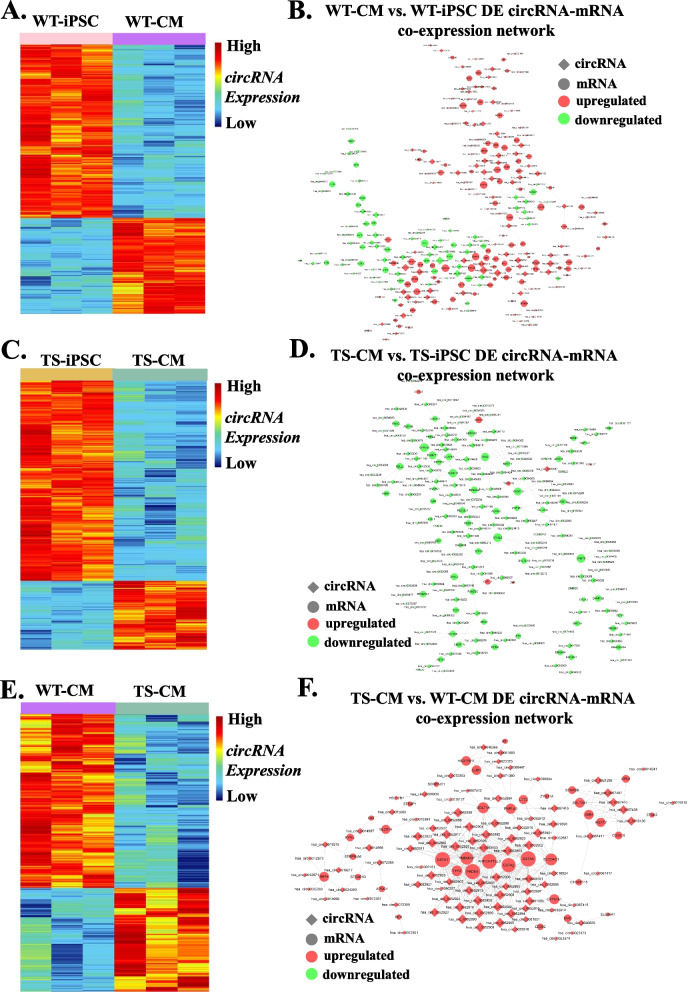
Identification of DE circRNAs and co-expression network analysis. **A** Heatmap showing the DE circRNAs in WT-CM versus WT-iPSC. **B** Co-expression network of DE circRNA-mRNA in WT-CM versus WT-iPSC. **C** Heatmap showing the DE circRNAs in TS-CM versus TS-iPSC. **D** Co-expression network of DE circRNA-mRNA in TS-CM versus TS-iPSC. **E** Heatmap showing the DE circRNAs in TS-CM versus WT-CM. **F** Co-expression network of DE circRNA-mRNA in TS-CM versus WT-CM

Also, co-expression networks were constructed according to the correlation analysis of the DE circRNAs and mRNAs in the group pairwise comparison (Fig. 6A, C and E). There were 251, 392 and 343 circRNA-mRNA co-expression pairs identified for WT-CM versus WT-iPSC, TS-CM versus TS-iPSC and TS-CM versus WT-CM, respectively (Fig. 6B, D and F).

The co-expression networks implied complex regulating relationships between lnc/circRNAs and mRNAs. One lnc/circRNA could regulate multiple genes in different ways while one gene could be regulated by multiple lnc/circRNAs.

### X-inactivation escaping lnc/circRNAs regulate autosomal genes via ceRNA network

**Fig. 7:**
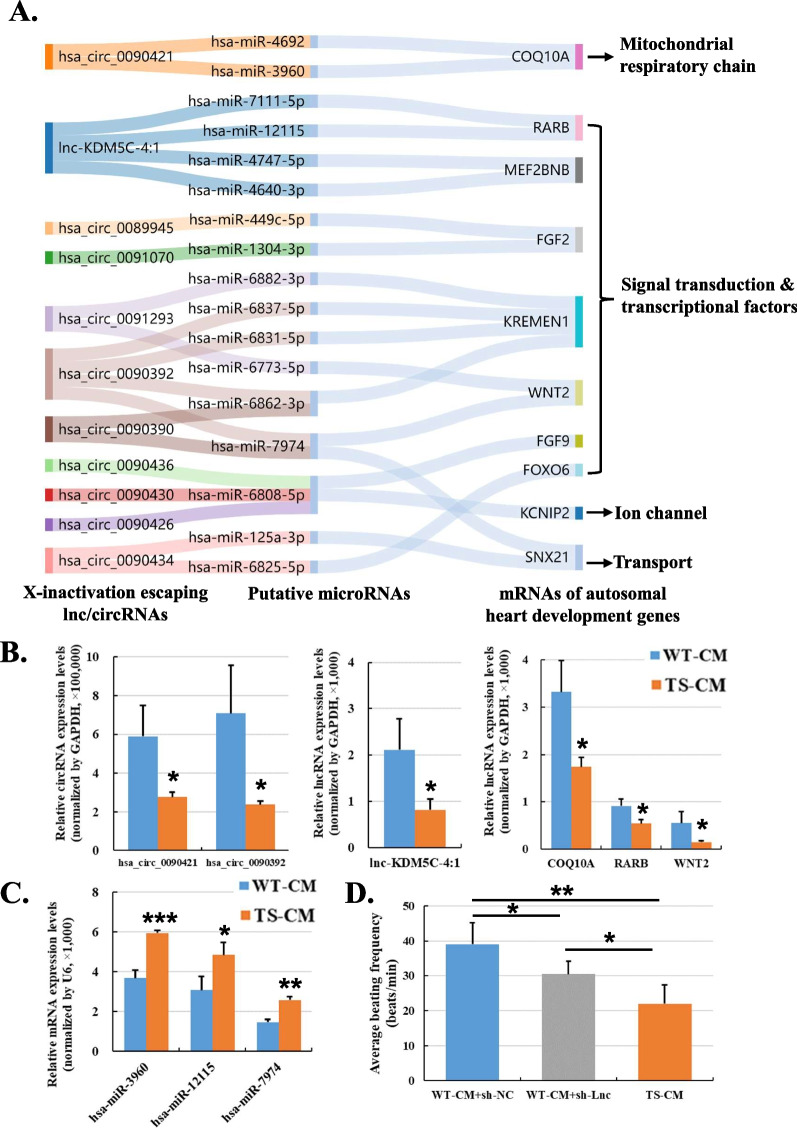
Identification and verification of X-linked non-coding RNA to autosomal coding RNA regulatory circuits. **A** A Sankey diagram showing putative lncRNAs/circRNA-microRNA-mRNA regulatory circuits of X-linked lnc/circRNAs to autosomal mRNAs relevant with heart development in TS-CMs. **B** Verification of the expression levels of representative lncRNAs, circRNAs and mRNAs from the putative regulatory circuits in WT- and TS-CMs by qPCR. **C** Verification of the expression levels of representative microRNAs from the putative regulatory circuits in WT- and TS-CMs by qPCR. **D** Average beating frequency of CMs at day 8. A total of three batches of CMs were generated from iPSCs, and four CM clusters of each batch were evaluated. Sh-NC, shRNA-Nonsense-Control and sh-Lnc, shRNA-lnc-KDM5C-4:1. Error bars indicate s.d.; *P < 0.05; **P < 0.01; ***P < 0.001

In view of the complex lnc/circRNA-mRNA correlations, we further predicted the miRNA targeting these DE mRNAs, lncRNAs and circRNAs and performed ceRNA network analysis to identified the putative “lnc/circRNA-miRNA-mRNA” regulatory circuits. There were hundreds of putative circuits identified, and we focused on those involved lnc/circRNAs whose host genes are X-inactivation escaping genes and mRNAs associated with cardiovascular development, CM functions or mitochondrial functions. Finally, we have identified that 11 X-inactivation escaping lnc/circRNAs, such as lnc-KDM5C-4:1 hosted by KDM5C, hsa_circ_0090421 hosted CDK16 and hsa_circ_0090392 hosted by UBA1, might regulate autosomal heart development genes via ceRNA network (Fig. 7A). The majority of these autosomal genes, such as RARB, WNT2, FGF2 and FGF9, were associated with the retinoic acid (RA) and WNT signals, which are important signaling pathways regulating CM differentiation. The other mRNAs were associated with different aspects of CM functions. For example, COQ10A was associated with mitochondrial respiratory chain, KCNIP2 was associated with ion channels and SNX21 was associated with intracellular protein trafficking in CMs. Furthermore, we have selected several putative circuits and verified the expression levels of the lnc/circRNAs, miRNAs and mRNAs by qPCR (Fig. 7B and C ) (Additional file 8: Table S5).

For further validation of the functional roles of the newly identified X-inactivation escaping lnc/circRNAs, lnc-KDM5C-4:1 was chosen for a loss-of-function assay. WT-iPSC lines were transduced with lentiviral vectors expressing shRNA-NC and shRNA-lnc-KDM5C-4:1, respectively. Then, the transduced lines underwent CM induction together with the TS-iPSC lines as negative controls. The beating frequencies of the CM lines were evaluated on day 8. The results showed that knockdown of lnc-KDM5C-4:1 in WT-CMs significantly reduced the beating frequency, though not as much as that of TS-CMs (Fig. 7D; Additional file 9: Video S3, Additional file 10: Video S4 and Additional file 11: Video S5). Hence, reduction of lnc-KDM5C-4:1 expression might partly tribute to impaired CM differentiation in TS.

## Discussion

In this study, we have presented an integrated approach using both iPSC and high-throughput transcriptomics technologies to investigate the genotype–phenotype relationships in CM differentiation of TS patients. In our and other groups’ of the previous studies, a variety of iPSC lines with sex chromosome aberrations have been generated to serve as excellent cellular models [14–18] to deepen our understandings of molecular network effects caused by supernumerary or reduced number of sex chromosomes [19–22]. Notably, the differentiation of patient-specific iPSCs into specific cell lineages has already proven valuable to study human diseases “in a Petri dish” and to develop novel therapeutic approaches [23–26]. Many studies have differentiated TS-iPSC lines into primordial germ cell-like cells, neural cells and bone cells to study the infertility, neurological deficits and skeleton abnormalities [27–30]. However, there is a lack of study on heart diseases using CMs derived from TS-iPSC lines. A risk assessment for TS patients has shown that 41% of the excess mortality is due to heart diseases [31]. Hence, we conducted this study to address the urgent need of illustrating the potential molecular mechanisms underlying heart diseases of TS patients.

The important regulatory functions of ncRNAs in the differentiation process of CMs have drawn great attentions for exploiting ncRNAs as novel therapeutic strategies of cardiomyopathies. For instance, CARMEN, a lncRNA associated with super-enhancers in the human myocardium, primarily controls the differentiation and homeostasis of CMs. Knockdown of CARMEN would inhibit further differentiation of cardiac progenitor cells into mature CMs [32]. HoxBlinc activates the HoxB gene through a direct interaction with the Set1/MLL complex thereby enabling the promotion of mesodermal cell differentiation [33]. Mouse embryos lacking Fendrr show a significant upregulation of Gata6 and Nkx2.5 expression levels, accompanied by a dramatic decrease in PRC2 occupancy, which affects the differentiation of embryonic stem cells to mesodermal cells [34]. Braveheart (Bvht) is a lncRNA found in mice associated with cardiac development, mainly as an upstream regulator of cardiovascular function, and has a very important role in the activation of the cardiovascular core gene network. It has also been shown that Bvht may co-regulate the cardiac gene expression program with PRC2 [35]. Regulated by the transcriptional repressor TBX2, TTN-AS1 has been revealed to be present at high expression levels during cardiac differentiation [36]. ECRAR has been reported to achieve promotion of myocardial regeneration and induction of cardiac function recovery after myocardial infarction [37].

Additionally, the involvement of circRNAs in CM proliferation and regeneration processes has also been highlighted [38]. Si et al*.* found an overexpression of circHipk3 in mice with myocardial infarction, demonstrating that circHipk3 promotes myocardial regeneration by promoting CM proliferation, maintaining cardiac function and reducing myocardial fibrosis by sponging miR-133 [39]. CircNfix has a negative regulatory role in CM proliferation and differentiation. CircNfix overexpression tends to reduce CM proliferation, whereas knockdown of circNfix promotes CM proliferation and angiogenesis [40]. CircTTN, a circRNA highly expressed in CMs, promotes proliferation and differentiation of adult CMs by competitively combining miR-432 and inhibiting the IGF2/PI3K/AKT signaling pathway [36, 41].

In our study, we have identified substantial alterations in expression levels of mRNA, lncRNA and circRNA within TS-CMs. Gene annotation enrichment analysis showed that the DE mRNAs were enriched of heart development genes. Further ceRNA network analysis has revealed that dysregulation of genes on autosomes could be possibly mediated by altered dosages of lnc/circRNAs on the X chromosome. The dysregulated autosomal genes are relevant with many aspects of heart development. For instance, COQ10A is an important component of the mitochondrial respiratory chain. The reduced COQ10A level might weaken the mitochondrial energetic function and lead to lower beating frequency as well as increased mitochondrial copies induced by negative feedback [42]. This may explain the altered phenotypes of TS-CMs we observed. A number of dysregulated genes, such as RARB, WNT2, FGF2 and FGF9, are involved in retinoic acid (RA) and WNT signaling pathways that are reported to be pivotal for regulating CM differentiation [43]. The X chromosome lnc/circRNAs involved in the above putative regulatory circuits are mostly expressed from X-inactivation escaping genes. For examples, lnc-KDM5C-4:1 is expressed from the KDM5C gene, hsa_circ_0090421 from CDK16 and hsa_circ_0090392 from UBA1. These X-inactivation escaping genes themselves are also reported to be relevant with CM regulation. The KDM5C gene on X chromosome and its homologous gene KDM5D on Y chromosome are encoding chromatin modifiers and related to regulation of sex-specific CM differentiation [44]. The UBA1 gene is reported to regulate the cardiac sodium channel via ubiquitination [45]. Further loss-of-function assay demonstrated that knockdown of lnc-KDM5C-4:1 by shRNA in WT-iPSCs would result in significantly reduced beating frequency post-CM induction. It is consistent with the phenotype of lower beating frequency observed in TS-CMs, though not as much as that of TS-CMs.

On the other hand, there are several limitations of this study. Firstly, owing to a restricted budget, merely three TS patient-specific cell lines and three healthy donor-derived cell lines were examined by microarrays in this study. It would better examine a larger cohort to exclude the individual variations as much as possible. Secondly, due to a lack of specialized equipment, only common phenotypes were evaluated for our CMs, such as cell morphology, immunostaining of cell-type markers, beating frequency and mitochondrial amount. It would be of interest to further characterize the electrophysical and metabolic features as well. Thirdly, due to a shortage of funding, the miRNA profiles of the model cell lines were not examined by miRNA microarray separately. Thus, the “lnc/circRNA-miRNA-mRNA” regulatory circuits were predicted by the enrichment of miRNA target sites putatively rather than by miRNA expression data directly, which could increase the false-positive rate of the prediction. Therefore, it still needs to get more resources to deepen the investigation of the ceRNA networks and their connections with functional phenotypes in a larger cohort of TS samples.

To date, there is no cure or effective treatment for TS patients. Nevertheless, iPSC and high-throughput transcriptomics technologies that enable identification of novel therapeutic targets may offer TS patients a new hope. Our study has provided iPSC-derived CMs as promising cellular tools for developing future TS therapies in combination with other biomedical technologies at the cutting edge. Firstly, CRISPR-mediated genome editing technologies can be used in iPSC-based models to investigate disease mechanisms and to develop novel therapies [24]. Secondly, iPSC-CMs can incorporate with 3D bioprinting and 3D culture technologies to establish organoid models for studying heart diseases in TS patients [46, 47], which enable investigating the underlying disease mechanisms in a microenvironment more relevant to that in vivo. Additionally, single-cell RNA-seq technology and pseudotemporal analysis can be used to illustrate a high-resolution dynamic transcriptome regulatory network during CM differentiation of iPSCs and identify new therapeutic targets for heart diseases in TS patients [48–50]. Furthermore, iPSC-CMs can be used for transplantation [51] as well as for drug screening [52] to treat adult TS patients with heart diseases. Taken together, our TS-iPSC lines reported here hold the potential for advancing research and developing therapies for adult TS.

## Conclusion

In summary, by application of the high-throughput ceRNA expression profiling in CMs derived from TS-iPSC lines, we have revealed a genomewide ripple effect caused by X chromosome halpoinsufficiency at post-transcriptional level and identified a number of novel “lnc/circRNA-miRNA-mRNA” regulatory circuits for mitochondrial respiratory chain and heart development genes on autosomes mediated by X-inactivation escaping lnc/circRNAs.

### Supplementary Information

**Additional file 1**. Additional Figures. **Figure S1**. Karyotypes of the iPSC lines. Karyotypes of (**A**) the 3 WT-iPSC lines and (**B**) the 3 TS-iPSC lines. **Figure S2**. Correlation analyses of mRNA, lncRNA and circRNA expression profiles among the samples. (**A**) principal component analysis (PCA) and (**B**) Pearson correlation coefficient (PCC) analysis of mRNA expression profiles among the samples of WT-iPSC, WT-CM, TS-iPSC and TS-CM groups. (**C**) PCA and (**D**) PCC analyses of lncRNA expression profiles among the samples. (**E**) PCA and (**F**) PCC analyses of circRNA expression profiles among the samples.

**Additional file 2**. **Video S1**. Beating WT-CM on Day 8.

**Additional file 3**. **Video S2**. Beating TS-CM on Day 8.

**Additional file 4**. **Table S1**. Differentially expressed mRNA.

**Additional file 5**. **Table S2**. Differentially expressed lncRNA.

**Additional file 6**. **Table S3**. Differentially expressed circRNA.

**Additional file 7**. **Table S4**. Annotation enrichment analysis of DE mRNA.

**Additional file 8**. **Table S5**. Primers used in this study.

**Additional file 9**. **Video S3**. Beating WT-CM+sh-NC on Day 8.

**Additional file 10**. **Video S4**. Beating WT-CM+sh-Lnc on Day 8.

**Additional file 11**. **Video S5**. Beating TS-CM on Day 8.

## Data Availability

The microarray data of this study are available in the Gene Expression Omnibus (GEO) database under the accession no. GSE239758. The other data and materials used in this study are available from the corresponding authors under reasonable request.

## Abbreviations

CM: Cardiomyocyte
iPSC: Induced pluripotent stem cell
hESC: Human embryonic stem cell
mRNA: Messenger RNA
lncRNA: Long non-coding RNA
circRNA: Circular RNA
miRNA: MicroRNA
ceRNA: Competing endogenous RNA
shRNA: Short hairpin RNA
TS: Turner syndrome
WT: Wild-type
PBMC: Peripheral blood mononuclear cell
AP: Alkaline phosphatase
EB: Embryoid body
DE: Differentially expressed
