# Supplementary material for: Competing endogenous RNA network analysis of Turner syndrome patient-specific iPSC-derived cardiomyocytes reveals dysregulation of autosomal heart development genes by altered dosages of X-inactivation escaping non-coding RNAs
Source: Stem Cell Res Ther. 2023 Dec 20;14:376. doi: 10.1186/s13287-023-03601-3 (PMC10734062; doi:10.1186/s13287-023-03601-3)

Supplementary Figure Legends

Supplementary Figure S1. Karyotypes of the iPSC lines. Karyotypes of **(A)** the 3 WT-iPSC lines and **(B)** the 3 TS-iPSC lines.

Supplementary Figure S2. Correlation analyses of mRNA, lncRNA and circRNA expression profiles among the samples. (A) principal component analysis (PCA) and **(B)** Pearson correlation coefficient (PCC) analysis of mRNA expression profiles among the samples of WT-iPSC, WT-CM, TS-iPSC and TS-CM groups. **(C)** PCA and **(D)** PCC analyses of lncRNA expression profiles among the samples. **(E)** PCA and **(F)** PCC analyses of circRNA expression profiles among the samples.

Supplementary Figure S1.

A.

WT1-iPSC

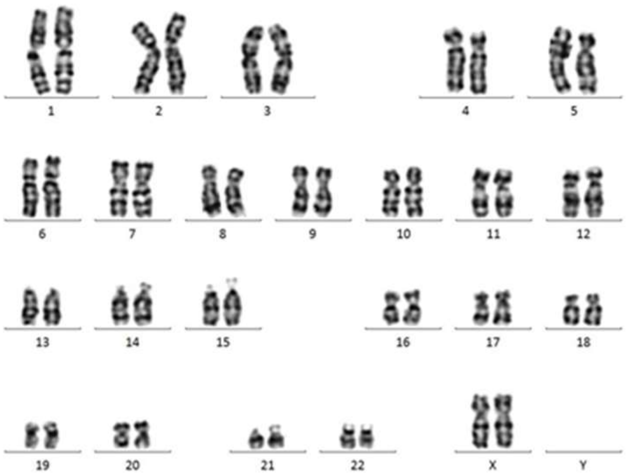

WT2-iPSC

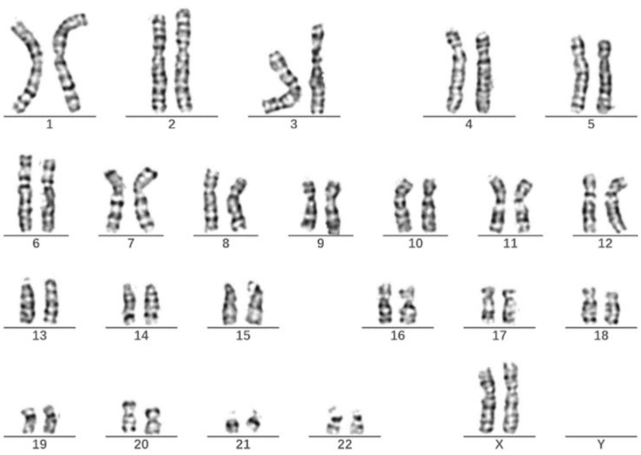

WT3-iPSC

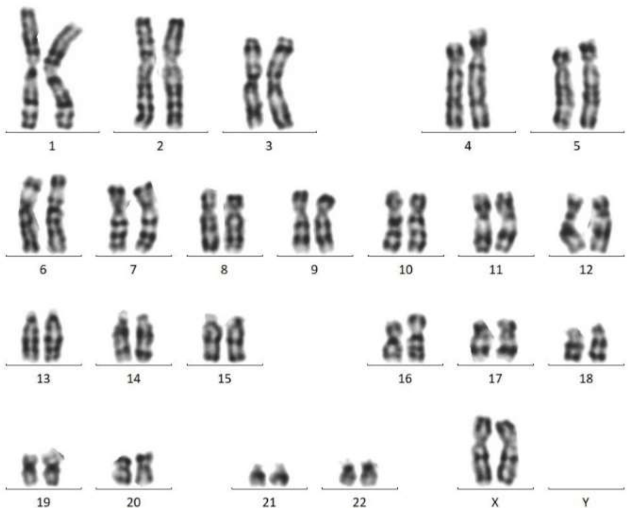

B.

TS1-iPSC

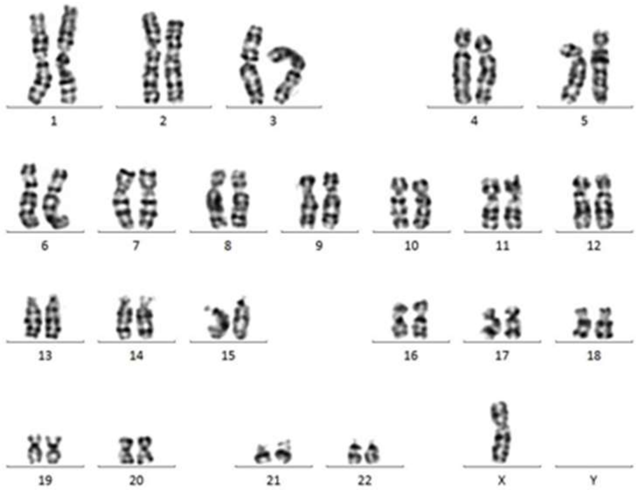

TS2-iPSC

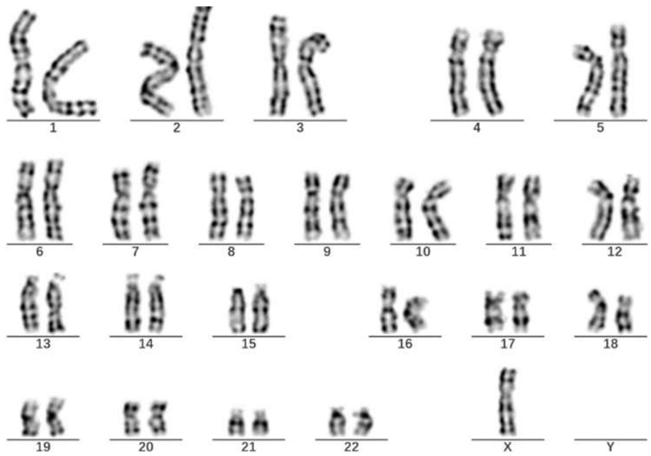

TS3-iPSC

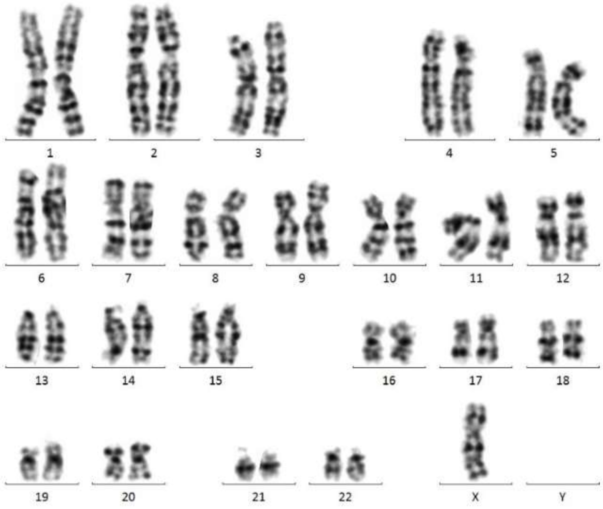

Supplementary Figure S2.

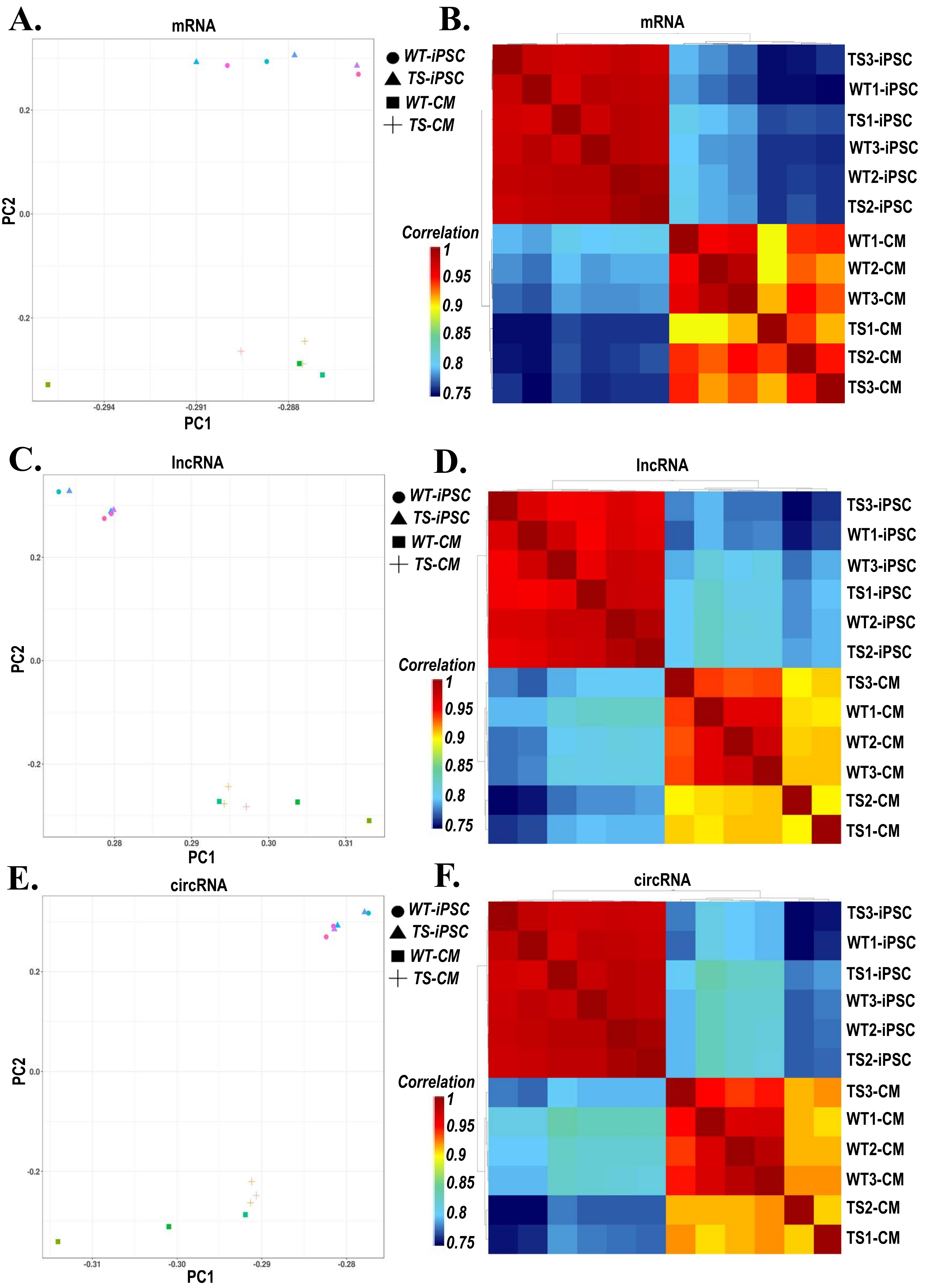

Supplement: Supplementary file 1 — Additional file 1. Additional Figures. Figure S1. Karyotypes of the iPSC lines. Karyotypes of (A) the 3 WT-iPSC lines and (B) the 3 TS-iPSC lines. Figure S2. Correlation analyses of mRNA, lncRNA and circRNA expression profiles among the samples. (A) principal component analysis (PCA) and (B) Pearson correlation coefficient (PCC) analysis of mRNA expression profiles among the samples of WT-iPSC, WT-CM, TS-iPSC and TS-CM groups. (C) PCA and (D) PCC analyses of lncRNA expression profiles among the samples. (E) PCA and (F) PCC analyses of circRNA expression profiles among the samples. [file 13287_2023_3601_MOESM1_ESM.pdf]
