# Supplementary material for: Competing endogenous RNA network analysis of Turner syndrome patient-specific iPSC-derived cardiomyocytes reveals dysregulation of autosomal heart development genes by altered dosages of X-inactivation escaping non-coding RNAs
Source: Stem Cell Res Ther. 2023 Dec 20;14:376. doi: 10.1186/s13287-023-03601-3 (PMC10734062; doi:10.1186/s13287-023-03601-3)
Supplement: Supplementary file 8 — Additional file 8. Table S5. Primers used in this study. [file 13287_2023_3601_MOESM8_ESM.docx]

**Supplementary Table S5. Primers used in this study**

| **Genes** | **Sequences** |
| --- | --- |
| ***mRNA, lncRNA, circRNA expression assay*** | |
| GAPDH | F: 5' TGACTTCAACAGCGACACCCA 3' |
|  | R: 5' CACCCTGTTGCTGTAGCCAAA 3' |
| COQ10A | F: 5' GGATGTGGAAATGAGATGTGCTT 3' |
|  | R: 5' AGGCATATGGTGCTCCTACGA 3' |
| RARB | F: 5' GGGATAATTAGCAGGCTGGTCTAC 3' |
|  | R: 5' AGGCATATGGTGCTCCTACGA 3' |
| WNT2 | F: 5' AAATAGCTACTTCTCGTCTTCTTGAAAAA 3' |
|  | R: 5' GATACCAGATGCATGTAATGAAATATCC 3' |
| lnc-KDM5C-4:1 | F: 5' TGCCTAGTTCCCTGTCGTTTG 3' |
|  | R: 5' GTGGAGGAGATGGCAGCAA 3' |
| hsa_circ_0090421 | F: 5' TCTTCGTCGATGCCTGACTC 3' |
|  | R: 5' TGAGGGGCTTGTCAAAGATGG 3' |
| hsa_circ_0090392 | F: 5' AAGCTGGGCAAGCAGAAGTATT 3' |
|  | R: 5' GCCGCTTCATTGCCTCAT 3' |
| ***microRNA expression assay*** | |
| rno-U6 | 5' TTCGTGAAGCGTTCCATATTTT 3' |
| hsa-miR-3960 | 5' GGCGGCGGCGGA 3' |
| hsa-miR-12115 | 5' TAGTGGAGCTGGGAGGCA 3' |
| hsa-miR-7974 | 5' AGGCTGTGATGCTCTCCTG 3' |
| ***Mitochondrial DNA copy number assay*** | |
| GAPDH | F: 5' GGATGATGTTCTGGAAGAGCC 3' |
|  | R: 5' AACAGCCTCAAGATCATCAGC 3' |
| 16S rRNA | F: 5' ACTTTGCAAGGAGAGCCAAA 3' |
|  | R: 5' TGGACAACCAGCTATCACCA 3' |
